# Comprehensive multi‐omics analysis of resectable locally advanced gastric cancer: Assessing response to neoadjuvant camrelizumab and chemotherapy in a single‐center, open‐label, single‐arm phase II trial

**DOI:** 10.1002/ctm2.1674

**Published:** 2024-04-29

**Authors:** Yuzhou Zhao, Danyang Li, Jing Zhuang, Zhimeng Li, Qingxin Xia, Zhi Li, Juan Yu, Jinbang Wang, Yong Zhang, Ke Li, Shuning Xu, Sen Li, Pengfei Ma, Yanghui Cao, Chenyu Liu, Chunmiao Xu, Zhentian Liu, Jinwang Wei, Chengjuan Zhang, Lei Qiao, Xuan Gao, Zhiguo Hou, Chenxuan Liu, Rongrong Zheng, Du Wang, Ying Liu

**Affiliations:** ^1^ Department of Surgical Oncology The Affiliated Cancer Hospital of Zhengzhou University, Henan Cancer Hospital Zhengzhou China; ^2^ Department of Medical Oncology The Affiliated Cancer Hospital of Zhengzhou University, Henan Cancer Hospital Zhengzhou China; ^3^ Department of Pathology The Affiliated Cancer Hospital of Zhengzhou University, Henan Cancer Hospital Zhengzhou China; ^4^ Department of Endoscopy Center The Affiliated Cancer Hospital of Zhengzhou University, Henan Cancer Hospital Zhengzhou China; ^5^ Department of Immunotherapy The Affiliated Cancer Hospital of Zhengzhou University, Henan Cancer Hospital Zhengzhou China; ^6^ Department of Radiology The Affiliated Cancer Hospital of Zhengzhou University, Henan Cancer Hospital Zhengzhou China; ^7^ Department of translational medicine Geneplus‐Beijing Institute Beijing China; ^8^ GenomiCare Biotechnology LA Co., Ltd. Shanghai China; ^9^ Jiangsu Hengrui Pharmaceuticals Co., Ltd. Shanghai China

**Keywords:** chemotherapy, gastric cancer, molecular markers, neoadjuvant therapies, PD‐1 inhibitor

## Abstract

**Background:**

The current standard of care for locally advanced gastric cancer (GC) involves neoadjuvant chemotherapy followed by radical surgery. Recently, neoadjuvant treatment for this condition has involved the exploration of immunotherapy plus chemotherapy as a potential approach. However, the efficacy remains uncertain.

**Methods:**

A single‐arm, phase 2 study was conducted to evaluate the efficacy and tolerability of neoadjuvant camrelizumab combined with mFOLFOX6 and identify potential biomarkers of response through multi‐omics analysis in patients with resectable locally advanced GC. The primary endpoint was the pathological complete response (pCR) rate. Secondary endpoints included the R0 rate, near pCR rate, progression‐free survival (PFS), disease‐free survival (DFS), and overall survival (OS). Multi‐omics analysis was assessed by whole‐exome sequencing, transcriptome sequencing, and multiplex immunofluorescence (mIF) using biopsies pre‐ and post‐neoadjuvant therapy.

**Results:**

This study involved 60 patients, of which 55 underwent gastrectomy. Among these, five (9.1%) attained a pathological complete response (pCR), and 11 (20.0%) reached near pCR. No unexpected treatment‐emergent adverse events or perioperative mortality were observed, and the regimen presented a manageable safety profile. Molecular changes identified through multi‐omics analysis correlated with treatment response, highlighting associations between HER2‐positive and CTNNB1 mutations with treatment sensitivity and a favourable prognosis. This finding was further supported by immune cell infiltration analysis and mIF. Expression data uncovered a risk model with four genes (*RALYL*, *SCGN*, *CCKBR*, *NTS*) linked to poor response. Additionally, post‐treatment infiltration of CD8+ T lymphocytes positively correlates with pathological response.

**Conclusion:**

The findings suggest the combination of PD‐1‐inhibitor and mFOLFOX6 showed efficacy and acceptable toxicity for locally advanced GC. Extended follow‐up is required to determine the duration of the response. This study lays essential groundwork for developing precise neoadjuvant regimens.

## Graphical abstract

The combination of PD‐1‐inhibitor and mFOLFOX6 is effective and well‐tolerated in patients with locally advanced GC

HER2‐positive and CTNNB1 mutations are associated with treatment sensitivity and a favourable prognosis in patients with locally advanced GC.

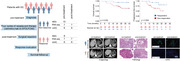

## 1 INTRODUCTION

Gastric cancer (GC) is a prevalent form of gastrointestinal malignant and one of the most deadly cancers globally.^1^ Common approaches for treating localized GC include endoscopic resection or surgery. However, most patients develop recurrence after resection. The UK MAGIC trial, the French FNCLCC/FFCD trial, the German FLOT4‐AIO trial, and the Chinese RESOLVE trial showed the clinical benefits of perioperative chemotherapy in patients with locally advanced GC.^2^, ^3^, ^4^, ^5^ Despite advances in treatment, outcomes for patients with GC remain unsatisfactory. Thus, multimodal treatment and understanding the mechanistic basis of therapeutic response are crucial to improve clinical practice.

Immune checkpoint inhibitors (ICIs) combined with chemotherapy have made a breakthrough in the treatment of advanced carcinoma, including GC. Studies including ATTRACTION‐2, KEYNOTE‐062, and CheckMate649 have shown promising results and are now regularly used for managing patients with advanced‐stage GC.^6^, ^7^, ^8^ Further exploration is still required to determine the safety and effectiveness of neoadjuvant immunotherapy in resectable advanced‐stage GC, due to the improved comprehension of the antitumour mechanism revealing potential synergistic effects when combined with chemotherapy.

Several clinical trials have explored the merits of neoadjuvant immunotherapy for locally advanced GC.^9^, ^10^ Nevertheless, the mechanisms by which neoadjuvant chemotherapy influences cancer signalling and impacts the tumour microenvironment remain not fully understood. A comprehensive understanding of these factors is essential for developing an effective multimodality treatment strategy. According to previous studies, the interaction between the tumour microenvironment and the genomic characteristics of the tumour affects the effectiveness of ICIs.^11^, ^12^, ^13^ However, the precise alterations in both the tumour microenvironment and cancer genome induced by combining ICIs with chemotherapy for GC remain uncertain. In this particular context, we carried out an investigative study of phase II, with a single‐arm design, to delve into the efficacy and tolerability of neoadjuvant camrelizumab (a PD‐1 inhibitor) plus mFOLFOX6 for locally advanced resectable GC. We also investigated the mechanism of action for chemoimmunotherapy and potential biomarkers for pathological response based on comprehensive multi‐omics analysis (genomics, transcriptomics, multiplex immunofluorescence).

## 2 METHODS

### 2.1 Trial design and treatments

Patients who met the inclusion criteria were 18−73 years old and were diagnosed with a locally advanced gastric and gastroesophageal junction adenocarcinoma (GA/GEJA) at a clinical‐stage ≥ cT2 and/or positive lymph nodes (cN^+^) confirmed with contrast‐enhanced CT/MRI chest abdomen pelvis and ultrasound gastroscopy, with no clinical evidence of distant metastases according to the 8th edition of the American Joint Committee on Cancer staging system.^14^ To be eligible, patients must have an Eastern Cooperative Oncology Group (ECOG) performance status (PS) of 0−2, an anticipated survival time of at least 3 months, and sufficient organ function. Key exclusion criteria were any prior systemic anticancer therapy, clinically significant previous or concurrent malignancies within 5 years before enrollment, grade 3 or worse hemorrhagic events within 4 weeks before enrollment, or the possibility of haemorrhage or fistula caused by tumour infiltration of the neighbouring organs (trachea or main aorta), central nervous system metastases, clinical signs of partial or complete bowel obstruction, or vaccination within 4 weeks before enrollment. They were administered four cycles of preoperative camrelizumab plus mFOLFOX6 and then proceeded to surgery if deemed to be resectable. An intravenous dose of 200 mg camrelizumab was given on the first day of every two‐week cycle. The mFOLFOX6 chemotherapy included oxaliplatin at 85 mg/m^2^ and leucovorin at 400 mg/m^2^, followed by 5‐fluorouracil at 400 mg/m^2^ and then 2.4 mg/m^2^ as 46 h of continuous intravenous infusion on the first day of each cycle. Patients completing neoadjuvant therapy were assessed for eligibility for surgery. Surgical resection was performed according to the institutional standard procedures. Postoperative adjuvant therapy was specified during the study period. Patients were offered another 4 or 6 (3‐ or 2‐week cycle) courses of camrelizumab plus mFOLFOX6 postoperatively, followed by camrelizumab monotherapy for up to 1 year.

### 2.2 Clinical trial outcomes

The primary endpoint was the pathological complete response (pCR) rate. The pCR rate was the proportion of patients with a tumour regression score of 0, which describes no residual disease including lymph nodes according to a simplified four‐tiered tumour regression grading system recommended by the College of American Pathologists.^15^ Secondary endpoints included the R0 rate, near pCR rate, progression‐free survival (PFS), disease‐free survival (DFS), overall survival (OS), and safety. The R0 rate was determined as the proportion of patients who had no remaining tumour cells at the primary cancer site and negative lymph nodes by histology. The near pCR rate was the proportion of patients with a tumour regression score of 1. The PFS was the interval from the first dose to disease relapse, progression, or death. The DFS was the interval from the baseline imaging evaluation after surgery to disease recurrence or death in patients who were free of disease post‐surgery. The OS was the interval from the first dose to all‐cause death. Treatment‐emergent adverse events (TEAEs) were assessed using the Common Terminology Criteria for Adverse Events, version 4.0. Immune‐related AEs (irAEs) were TEAEs with a potential immunological basis that requires close monitoring or potential intervention according to the Guidelines for the management of immunotherapy‐related toxicities.^16^, ^17^ Exploratory endpoints aimed to investigate the mechanism of action for chemoimmunotherapy and potential biomarkers for pathological response in GA/GEJA using available tumour samples.

### 2.3 Immunohistochemical and in situ hybridization

Pretreated tissues were used to determine the state of EBV, PD‐L1, HER2, and MMR. PD‐L1 expression was evaluated by immunohistochemical (IHC) utilizing a rabbit monoclonal antibody (Clone SP263, Ventana). The combined positive score (CPS) was calculated as the fraction of all PD‐L1 positive cells (tumour and immune cells) to the total amount of viable tumour cells. HER2 status was evaluated by IHC using a rabbit monoclonal antibody (Roche Diagnostics) in combination with FISH utilizing a Ventana HER2 Dual ISH DNA Probe Cocktail (Roche Diagnostics). HER2 positive (HER2^+^) was defined as IHC 3^+^ or IHC 2^+^ plus FISH positive. MMR status was assessed with the VENTANA MMR IHC Panel consisting of VENTANA anti‐MLH1 (clone M1), VENTANA anti‐PMS2 (clone A16‐4), VENTANA anti‐MSH2 (clone G219‐1129), and VENTANA anti‐MSH6 (clone SP93). dMMR was defined as the lack of expression of one or more proteins, while MMR proficient was the maintained expression of all four proteins. EBV status was evaluated by chromogenic in situ hybridization using an EBV‐specific probe (INFORM EBER probe, Ventana‐Roche Diagnostics).

### 2.4 Tumour mutational burden

Tumour mutational burden (TMB) was determined as the amount of non‐synonymous mutations. The clonal TMB was determined as the number of non‐synonymous mutations of which frequencies are more than 50% of the maximum mutation frequency. Tumour neoantigen burden was determined as the number of neoantigens per Mb in the genome region.

### 2.5 Genome pathway analysis

When gene alterations were detected within the gene set of a particular pathway in a sample, it was considered that the corresponding pathway in the sample had been altered. The corresponding intergroup pathway differences were determined by Fisher's exact test.

### 2.6 Differential gene expression analysis

Raw reads were evaluated for quality using FastQC followed by quality and adapter trimming with TrimGalore. HISAT2 was applied to align the clean reads of mRNA to the human reference genome hg19, and StringTie carried out transcript assembly and quantification. Differential mRNAs were identified with the absolute value of log_2_ (fold change) > 1 and *P*‐value < 0.05 by the DESeq2 package. A volcano plot of differentially expressed genes was generated by the visuz module of bioinfokit toolkit (https://doi.org/10.5281/zenodo.3965241) in Python.

### 2.7 Unsupervised hierarchical clustering of expression data

The standard deviation (SD) of DESeq2 normalized gene expression was calculated, and genes were ranked by decreasing SD across all samples, and only the top 1000 most variable coding genes were used for clustering. Unsupervised hierarchical clustering was performed using the seaborn (version 0.11.2) package and visualized by matplotlib in Python. Genes were ranked by decreasing standard deviation across all samples, and only the top 1000 most variable coding genes were used for clustering. PCA was then performed using the prcomp function in R to project samples into a two‐dimensional space. PCA plots were generated using the first two principal components and sample points were colored based on their genomic or clinical features.

### 2.8 Gene set enrichment analysis

Gene set enrichment analysis (GSEA)^18^ of the differentially expressed genes was performed using a pre‐rank method in the GSEApy (version 0.10.7) package in Python, with *n* = 1000 permutations. The input gene list used in GSEA was sorted by the test statistic of differential expression from DESeq2. The hallmark gene sets were derived from the GSEA molecular signatures database (gsea‐msigdb.org).

### 2.9 Tumour‐infiltrating immune cells analysis

RNA‐seq reads were assembled using StringTie2 (version 1.3.5) and an expression matrix including FPKM (fragments per kilobase of transcript sequence per million mapped fragments) and TPM (transcripts per kilobase of transcript sequence per million mapped reads) was generated. TPM data was used to calculate immune cell infiltration using the Immunedeconv R package based on the Quantiseq method,^19^ and the relative infiltration level of 36 immune cell types in the tumour microenvironment for each patient was represented by an enrichment score, as well as an immune score and a microenvironment score.

### 2.10 Pathway enrichment analysis

Most frequently seen mutations, their distribution across two groups (responders and non‐responders/pre‐treatment and post‐treatment), and the significantly different mutation genes between the two groups were calculated and visualized using the MAFtools package in R.^20^ Using the “Oncogenic Pathways” module in MAFtools, we analyzed the enrichment of canonical oncogenic signalling pathways based on TCGA cohorts.^21^ We assigned mutated genes to oncogenic signalling pathways and computed the fractions of genes involved in each pathway.

### 2.11 CD8 T cells immunofluorescence staining

For assessment of CD8 T‐cell activity, immunofluorescence was performed on paired pre‐ and post‐treatment tumour samples from 12 patients (six responders and six non‐responders). Briefly, slides were deparaffinized and rehydrated before antigen retrieval. Endogenous peroxidase activity was blocked with 3% H_2_O_2_ at room temperature for 15 min. The slides were then incubated with a rabbit recombinant anti‐CD8 alpha antibody [CAL66] (ab237709, 1:1000; Abcam) overnight at 4°C, followed by horseradish peroxidase‐conjugated goat anti‐rabbit IgG at room temperature for 50 min in dark conditions. The slides were subsequently stained with CY3‐Tyramide (red, G1223, Servicebio). The nucleus was counterstained with DAPI (blue, G1012, Servicebio).

### 2.12 Public database analysis

TCGA GC and GSE84437 transcriptomal profiles were downloaded from the TCGA Data Portal (http://tcga‐data.nci.nih.gov/tcga/) and NCBI public data platform, respectively. We applied the sample gene‐set enrichment analysis (ssGSEA) algorithm to compute the risk score from the gene expression level. The stromal score, immune score, and estimated score for each patient were calculated using the “estimate” R package. IMvigor210 dataset (http://research‐pub.gene.com/IMvigor210CoreBiologies)^22^ were employed to determine the relationship between the risk score and cancer immunotherapies.

### 2.13 Statistical analysis

This was a descriptive, exploratory study with no hypotheses being tested. All the results were descriptive and the *P*‐values were nominal *P*‐values. The number and proportion of patients with pCR, near‐pCR rate, and R0 rate were reported. The 95% confidence interval of pCR rate was estimated by using the Clopper–Pearson method. Time‐to‐events data were analyzed by the Kaplan–Meier method, and the 95% confidence intervals for time‐to‐events were calculated by using the Brookmeyer and Crowley method. The post hoc pathological response (pCR+ near pCR) subgroup analysis included primary tumour location (GEJ or gastric), age (<65 or ≥65 years), clinical T staging (cT3 or cT4), sex (male or female), ECOG PS (0 or 1), HER2 status (negative or positive), PD‐L1 CPS (<1 or ≥1), PD‐L1 CPS (<5 or ≥5), and PD‐L1 CPS (<10 or ≥10). Fisher's exact test was used for categorical data comparisons. Pre‐ and post‐treatment differences were analyzed using paired *t*‐test. The Mann–Whitney *U* test was used for continuous data comparisons unless specified otherwise. Statistical analysis and data visualization were performed with SAS (version 9.4), R (version 4.0.3), and the Seaborn package in Python (version 3.7). All statistical analyses were two‐sided.

## 3 RESULTS

### 3.1 Overview of patient cohort

**FIGURE 1:**
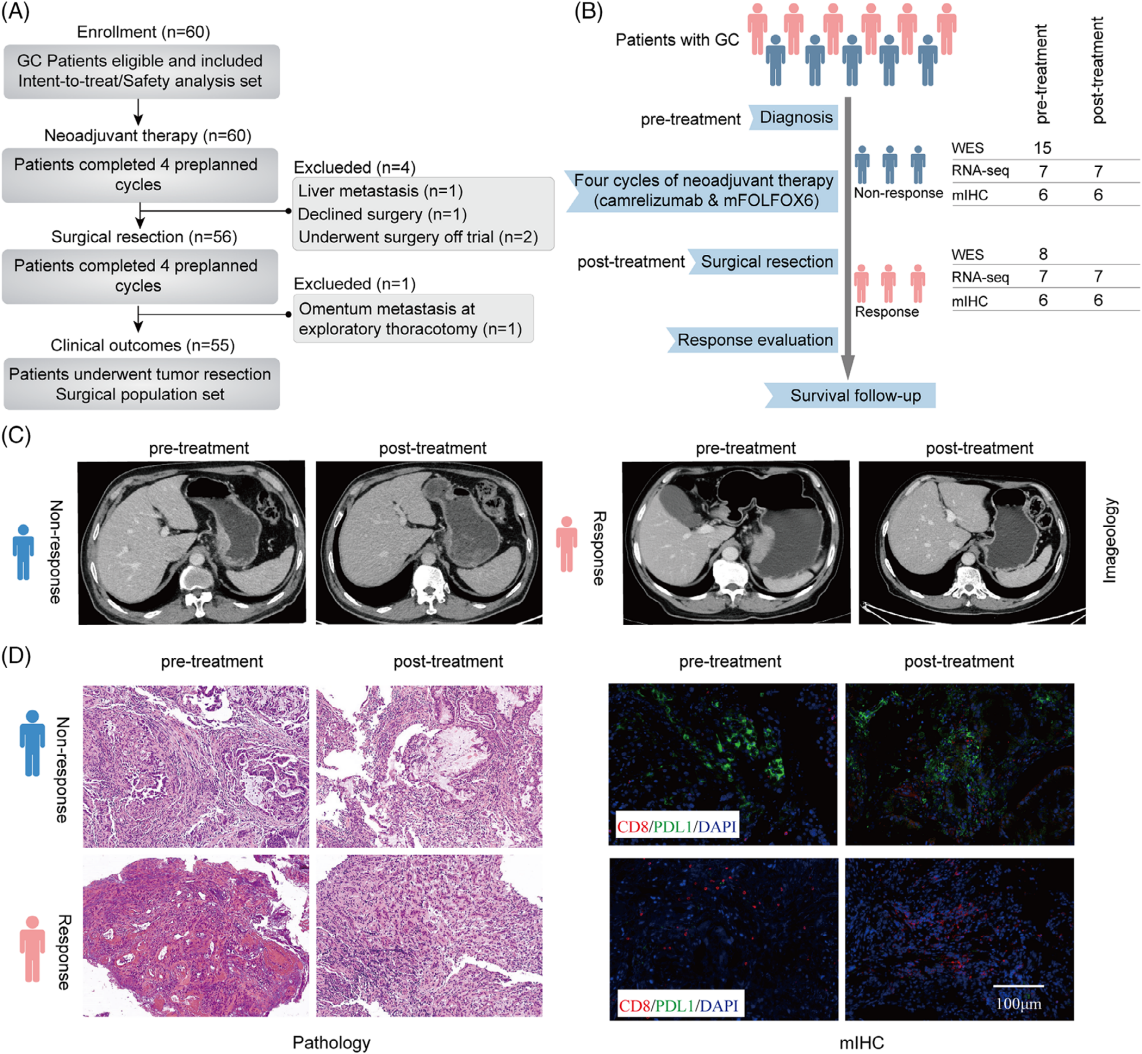
Study overview. (A) Study flow chart. (B) Overview of the study. The representative radiological (C) and pathological images (D) from responsive and nonresponsive patients.

Between July 2019 and February 2021, 60 patients were screened for eligibility and subsequently enrolled in the study. All patients completed four cycles of neoadjuvant therapy with camrelizumab in combination with mFOLFOX6. Among the enrolled patients, two individuals were unable to undergo surgery due to metastasis, one patient declined surgery, and two patients underwent surgery outside the trial's parameters. Consequently, a total of 55 patients underwent gastrectomy as part of the study (Figure 1A). The majority of patients were male (71.7%) and had clinical stage III cancer involvement (96.7%). Out of the 60 patients, 38 (63.3%) had primary tumours located at the GEJ and 32 (53.3%) had PD‐L1 CPS ≥ 1. Eight patients tested positive for HER2, four for EBV, and three for dMMR, with no overlap among them. The patients exhibited diverse histological traits and tumour distributions (Table 1). We conducted whole exome sequencing (WES), RNA‐seq, and multiplex immunofluorescence (mIF) on available tumour samples from both responders and non‐responders either at baseline or post‐surgery (Figure 1B, C, D). We then analyzed the association between the multi‐omics findings and clinical outcomes.

**TABLE 1:** Baseline patient characteristics.

Variables	Total (*n* = 60)
Median age, years (range)	58 (29, 72)
Sex, *n* (%)	
Male	43 (71.7)
Female	17 (28.3)
ECOG performance score, *n* (%)	
0	36 (60.0)
1	24 (40.0)
Histologic type, *n* (%)	
Adenocarcinoma	60 (100.0)
Signet‐ring cell carcinoma^a^	6 (10.0)
Primary tumour location, *n* (%)	
Gastro‐oesophagal junction	38 (63.3)
Gastric	22 (36.7)
Histologic grade, *n* (%)	
G1	0 (0.0)
G2	2 (3.3)
G3	31 (51.7)
GX	27 (45.0)
Clinical T stage, *n* (%)	
cT2	2 (3.3)
cT3	30 (50.0)
cT4	28 (46.7)
Clinical N stage, *n* (%)	
cN^+^	60 (100.0)
Clinical stage, *n* (%)	
II	2 (3.3)
III	58 (96.7)
PD‐L1 status, CPS, *n* (%)	
<1	19 (31.7)
≥1	32 (53.3)
1–5	13 (21.7)
5–10	8 (13.3)
≥10	11 (18.3)
Unknown	9 (15.0)
HER2 status, *n* (%)	
Positive	8 (13.3)
Negative	52 (86.7)
EBV status, *n* (%)	
Positive	4 (6.7)
Negative	47 (78.3)
Unknown	9 (15.0)
MMR status, *n* (%)	
dMMR	3 (5.0)
pMMR	54 (90.0)
Unknown	3 (5.0)

Abbreviations: CPS, combined proportion score; dMMR, MMR deficient; EBV, Epstein–Barr virus; ECOG, Eastern Cooperative Oncology Group; HER2, human epidermal growth factor receptor 2; MMR, mismatch repair; PD‐L1, programmed death ligand‐1; pMMR, MMR proficient.

^a^Of the 60 gastric adenocarcinomas, six were histologically signet‐ring cell carcinoma.

### 3.2 Patient characteristics and clinical outcomes

Out of the 60 patients, five (8.3%) achieved pCR (tumour regression grade [TRG] 0), and 11 (18.3%) achieved near pCR (TRG 1). Fifty‐one patients (85.0%) underwent R0 resection and 37 patients (61.7%) experienced tumour downstaging. In the surgical population set, the pCR rate was 9.1% (5/55), the near pCR rate was 20.0% (11/55), and the R0 resection rate was 92.7% (51/55) (three patients found omentum metastasis in postoperative pathology and one with positive margin; Table 2). A previous study reported that patients with TRG 0−1 achieved significantly better outcomes in terms of DFS and OS compared with those with TRG2 or TRG3.^23^ Therefore, patients with pCR or near pCR were defined as responders, while those with partial, poor, or no response were termed non‐responders. Due to postoperative omentum metastasis in three patients, 52 patients received adjuvant therapy. Responders received a median of 12 adjuvant therapy cycles (range 4−14), while non‐responders had a median of 10 cycles (range 2−13). Responders discontinued adjuvant therapy due to grade 3 immune‐associated transaminases increased (*n* = 1) and patient choice (*n* = 1), while non‐responders discontinued due to grade 3 nausea and grade 3 vomiting (*n* = 1) and disease progression (*n* = 7; Table S1).

**TABLE 2:** Pathological outcomes in the surgical population set.

	Total (*n* = 55)
Achieved R0 resection, *n* (%)	51 (92.7)
ypT stage, *n* (%)	
ypT0	5 (9.1)
ypT1	9 (16.4)
ypT2	7 (12.7)
ypT3	27 (49.1)
ypT4	7 (12.7)
ypN stage, *n* (%)	
ypN0	30 (54.5)
ypN1	7 (12.7)
ypN2	8 (14.5)
ypN3	10 (18.2)
ypTNM stage, *n* (%)	
0	5 (9.1)
I	13 (23.6)
II	20 (36.4)
III	14 (25.5)
IV	3 (5.5)
Tumour downstaging, *n* (%)	
Yes	37 (67.3)
No	18 (32.7)
Tumour regression, *n* (%)	
pCR	5 (9.1)
Near pCR	11 (20.0)
Partial response	26 (47.3)
Poor or no response	13 (23.6)

Abbreviation: pCR, pathological complete response.

**FIGURE 2:**
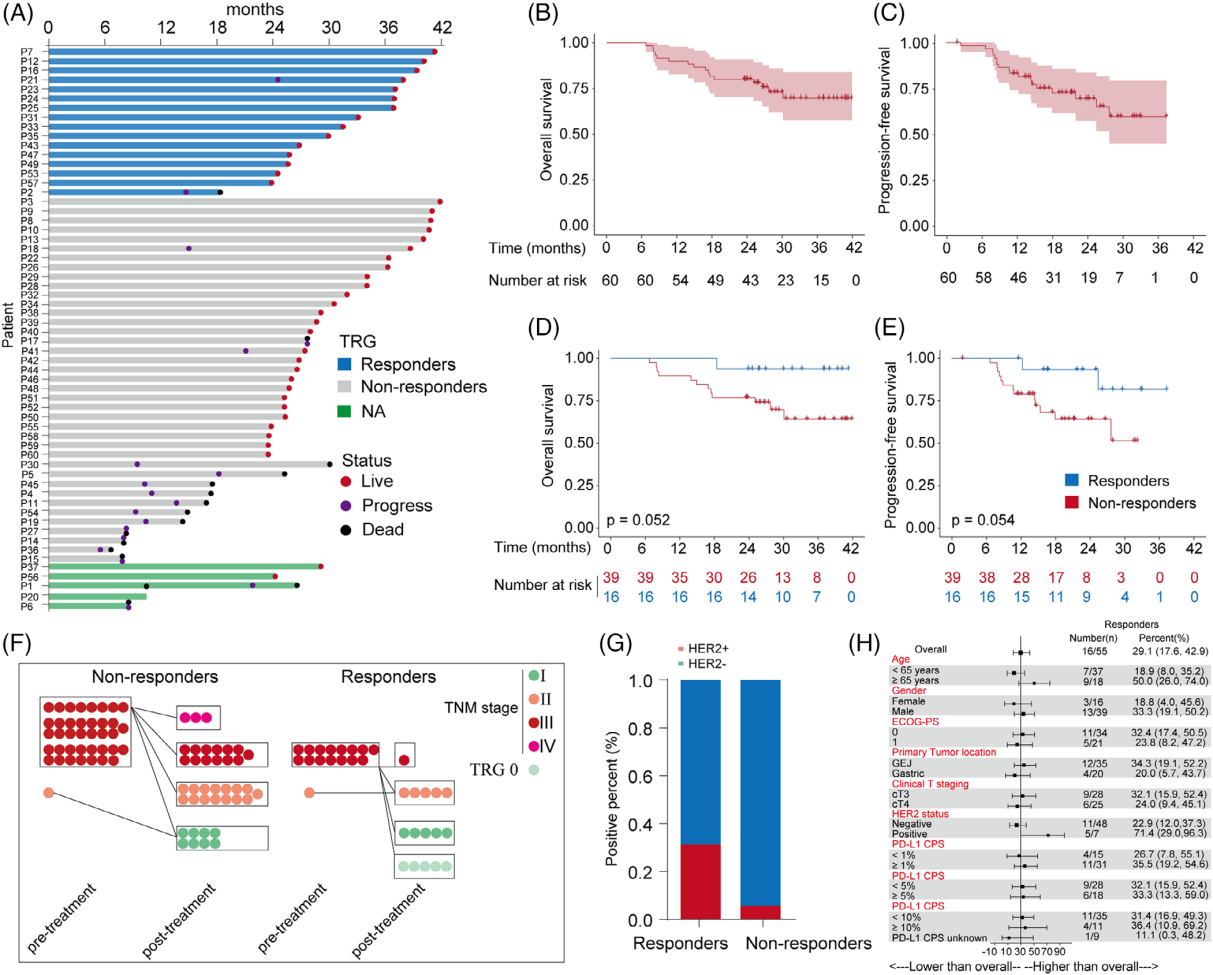
Efficacy and safety of neoadjuvant treatment. (A) Response and duration for the patients with neoadjuvant treatment. OS (B) and PFS (C) Kaplan–Meier survival curve for patients with neoadjuvant treatment. Kaplan–Meier survival analysis depicting the probability of OS (D) and PFS (E) stratified by treatment response status. (F) Comparative efficacy of neoadjuvant treatment for TNM staging. (G) Comparison of HER2 status between patients with response and non‐response. (H) Pathological response subgroup analysis based on baseline characteristics.

After a median follow‐up time of 26.5 months (IQR: 23.4–34.7), the median PFS and OS remained unreached at the data cutoff (14 January 2023, Figure 2A–C). The estimated one‐year PFS rate was 82% (95% CI, 88−100) and the two‐year PFS rate was 68% (95% CI 53−80). The estimated one‐ and two‐year OS rates were 90% (95% CI, 79−95) and 80% (95% CI, 67−88), respectively. The OS and PFS of responders demonstrated a statistically insignificant trend compared with that of non‐responders (Figure 2D, E). This discrepancy might be attributed to the relatively small patient number in this cohort. There were no unexpected TEAEs or perioperative mortality. Reactive cutaneous capillary endothelial proliferation was the most frequent irAE, occurring in 41 (68.3%) patients, and all were mild (grade 1−2). No grade 5 TEAEs were observed. The regimen presented a manageable safety profile (Table 3).

**TABLE 3:** Treatment‐emergent adverse events (TEAEs).

	TEAEs (*n* = 60)			
	Grade 1−2	Grade 3	Grade 4	Total
Immune‐related AEs				
RCCEP	41 (68.3)	0 (0.0)	0 (0.0)	41 (68.3)
Hypothyroidism	6 (10.0)	0 (0.0)	0 (0.0)	6 (10.0)
ALT increased	5 (8.3)	1 (1.7)	0 (0.0)	6 (10.0)
AST increased	4 (6.7)	1 (1.7)	0 (0.0)	5 (8.3)
Hyperthyroidism	4 (6.7)	0 (0.0)	0 (0.0)	4 (6.7)
Nonimmune‐related AEs				
White blood cells decreased	35 (58.3)	10 (16.7)	0 (0.0)	45 (75.0)
Neutropenia	29 (48.3)	14 (23.3)	3 (5.0)	43 (71.7)
Nausea	32 (53.3)	1 (1.7)	0 (0.0)	33 (55.0)
Anemia	16 (26.7)	0 (0.0)	0 (0.0)	16 (26.7)
Anorexia	16 (26.7)	0 (0.0)	0 (0.0)	16 (26.7)
Platelet count decreased	6 (10.0)	1 (1.7)	0 (0.0)	7 (11.7)
Hyponatremia	5 (8.3)	0 (0.0)	0 (0.0)	5 (8.3)
Hyperkalemia	4 (6.7)	0 (0.0)	0 (0.0)	4 (6.7)
Hypokalemia	4 (6.7)	0 (0.0)	0 (0.0)	4 (6.7)
Hematuria	1 (1.7)	0 (0.0)	0 (0.0)	1 (1.7)

Abbreviations: ALT, alanine aminotransferase; AST, aspartate aminotransferase; RCCEP, reactive cutaneous capillary endothelial proliferation.

### 3.3 Baseline clinical characteristics correlated with pathological response

Twenty‐three patients (59.0%) among non‐responders and 14 patients (87.5%) among responders experienced tumour downstaging (Figure 2F). Patients with HER2‐positive status demonstrated a superior response rate compared with those with HER2‐negative status (71.4% vs 22.9%). This difference achieved statistical significance (*P *= 0.0175) using Fisher's exact test. Furthermore, patients displaying HER2‐positive status exhibited a higher rate of response. (Figure 2G). Among patients with pCR, four out of five patients were PD‐L1 CPS ≥ 1, while one patient's PD‐L1 CPS status was unknown. Two patients were dMMR, one achieved pCR and the other achieved near pCR. Additionally, a post hoc subgroup analysis for pathological response (pCR + near pCR) was performed in the surgical population. The pathological response was slightly higher in the PD‐L1 CPS ≥ 1 group than in the PD‐L1 CPS < 1 group (35.5% vs. 26.7%) and was comparable between the two groups at a PD‐L1 CPS cut‐off of 5 and a cut‐off of 10 (33.3% vs 32.1%, 36.45% vs 31.4%; Figure 2H).

### 3.4 Genomic features associated with pathological response

**FIGURE 3:**
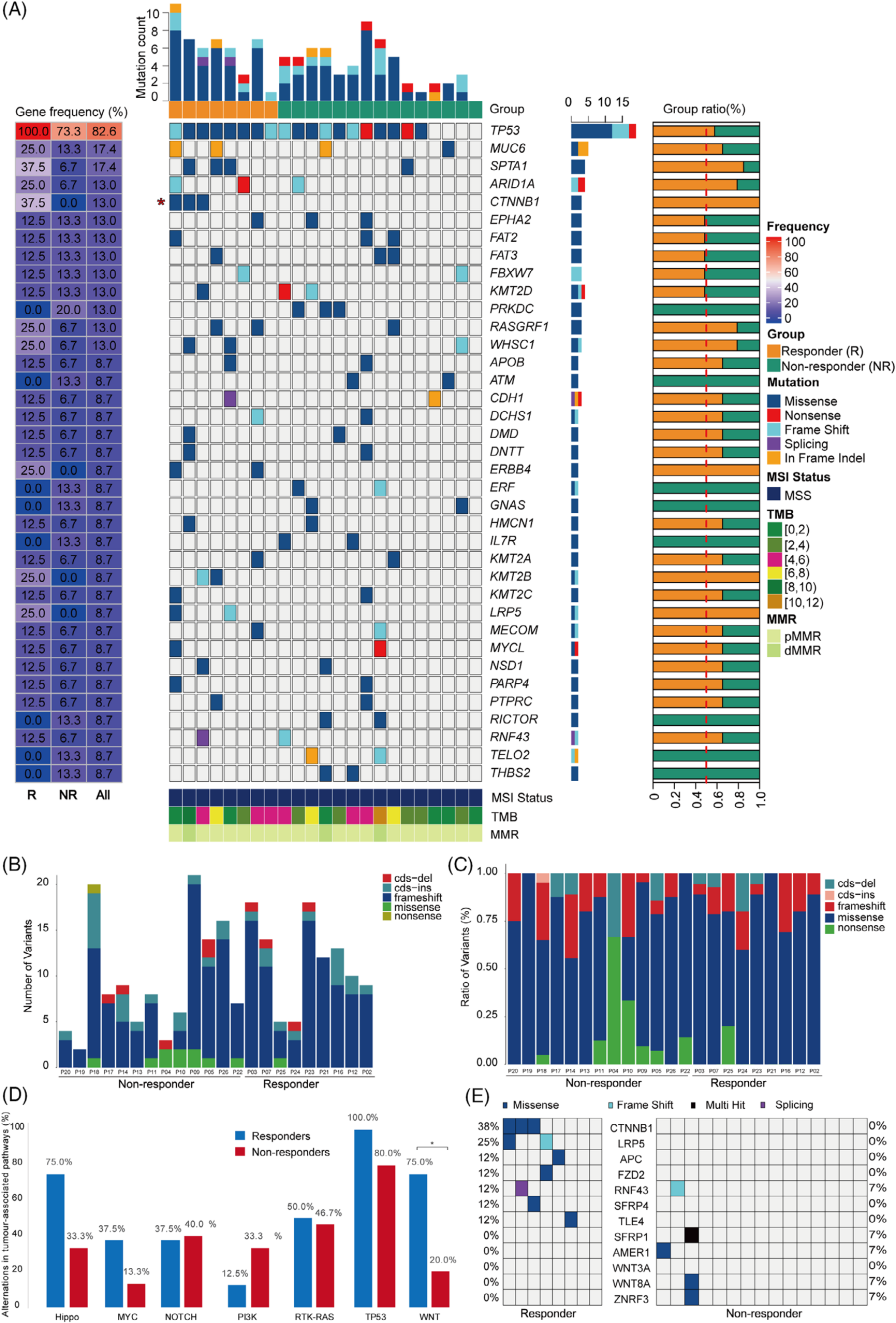
Genomic characteristics of tumours pre‐neoadjuvant therapy. Whole‐exome sequencing analysis was performed on baseline samples from eight responders and 15 non‐responders. (A) Heatmap showing functional mutations present in two or more patients. Number of variants (B) and ratio of variants (C) in tumours from pre‐treatment biopsies. (D) Comparison of tumour‐associated signalling pathways alterations between patients with response and non‐response. (E) Heatmap showing the mutational profile of the Wnt/β‐Catenin pathway. Each column represents a different sample and the rows represent genes.

We compared baseline tumour samples from eight responders and 15 non‐responders using WES to identify biomarkers that might predict pathological response. All patients were MSS. Two patients were EBV positive, one was a responder and the other was a non‐responder. HER‐2 positivity was detected in three patients, two were responders and the rest one was a non‐responder. The analysis of copy number variants (CNV) did not detect any notable distinctions (*P *< 0.05) between the groups of individuals who responded and those who did not respond (Figure S1). The median TMB was 4.60 mutations/Mb for responders and 3.17 mutations/Mb for non‐responders (*P *= 0.357). The median clonal TMB for responders was similar to that for non‐responders (0.35 and 0.9 for responders versus 0.60 and 0.3 for non‐responders with *P *= 0.746 and *P = *0.650, respectively; Figure 3A). Additionally, one patient exhibited a high TMB based on the KEYNOTE‐158′s definition of TMB high (≥10 mutations/Mb).^24^ Intriguingly, no significant differences were observed in variants in tumours from pre‐treatment biopsies between responders and non‐responders, which was due mainly to limited sample size (Figure 3B, C). Figure 3D showcases functional mutations, with the most frequently mutated genes being TP53, MUC6, SPTA1, and CTNNB1. Notably, three out of eight responders (37.5%) had catenin Beta 1 (CTNNB1) mutations compared with none in the non‐responders (0%) (*P *= 0.036, Figure 3E). Therefore, it is plausible to hypothesize that the CTNNB1 mutation may influence treatment response.

**FIGURE 4:**
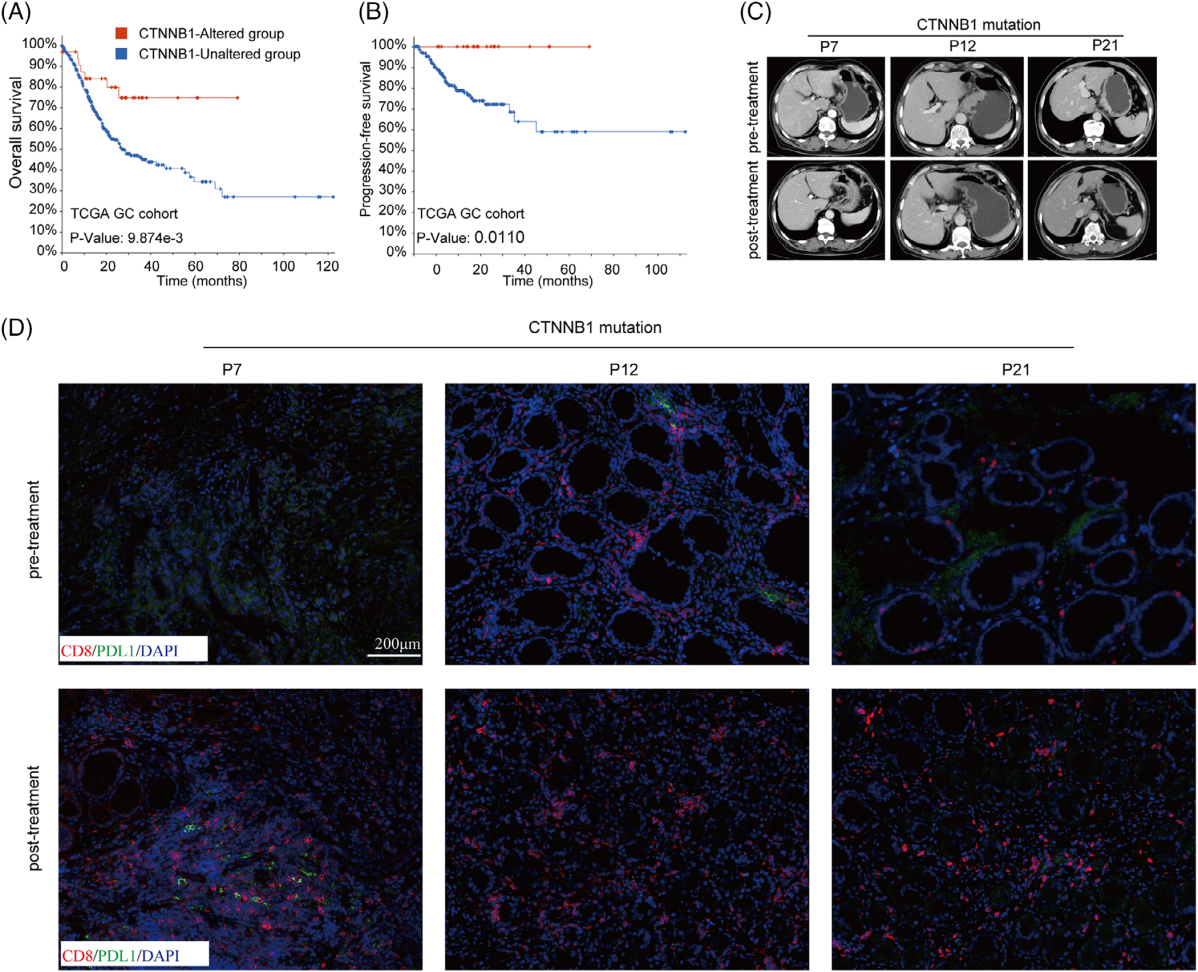
CTNNB1 mutation is associated with poor prognosis of GC patients. Kaplan–Meier curves of OS (A) and PFS (B) comparing the mutation status of CTNNB1 from GC patients in TCGA. The representative radiological (C) and pathological images (D) from CTNNB1‐mutation patients.

To support this hypothesis, we further examined the correlation between CTNNB1 mutation statuses and the OS of patients in the TCGA GC cohort. Our results indicated that individuals with the CTNNB1 mutation experienced a significantly prolonged survival duration (Figure 4A, B). To validate the impact of the CTNNB1 mutation on the tumour microenvironment during chemoimmunotherapy, we further assessed the tumour immune infiltration status using evidence from CD8+ T cells and PD‐L1 staining in patients with the CTNNB1 mutation via mIF (Figure 4C, D). The results of our study demonstrated a decrease in PD‐L1 expression, whereas a notable rise in CD8+ T lymphocytes was observed in all three patients after treatment. These findings imply that the CTNNB1 mutation could potentially regulate tumour immune infiltration.

### 3.5 Transcriptomic features associated with pathological response

**FIGURE 5:**
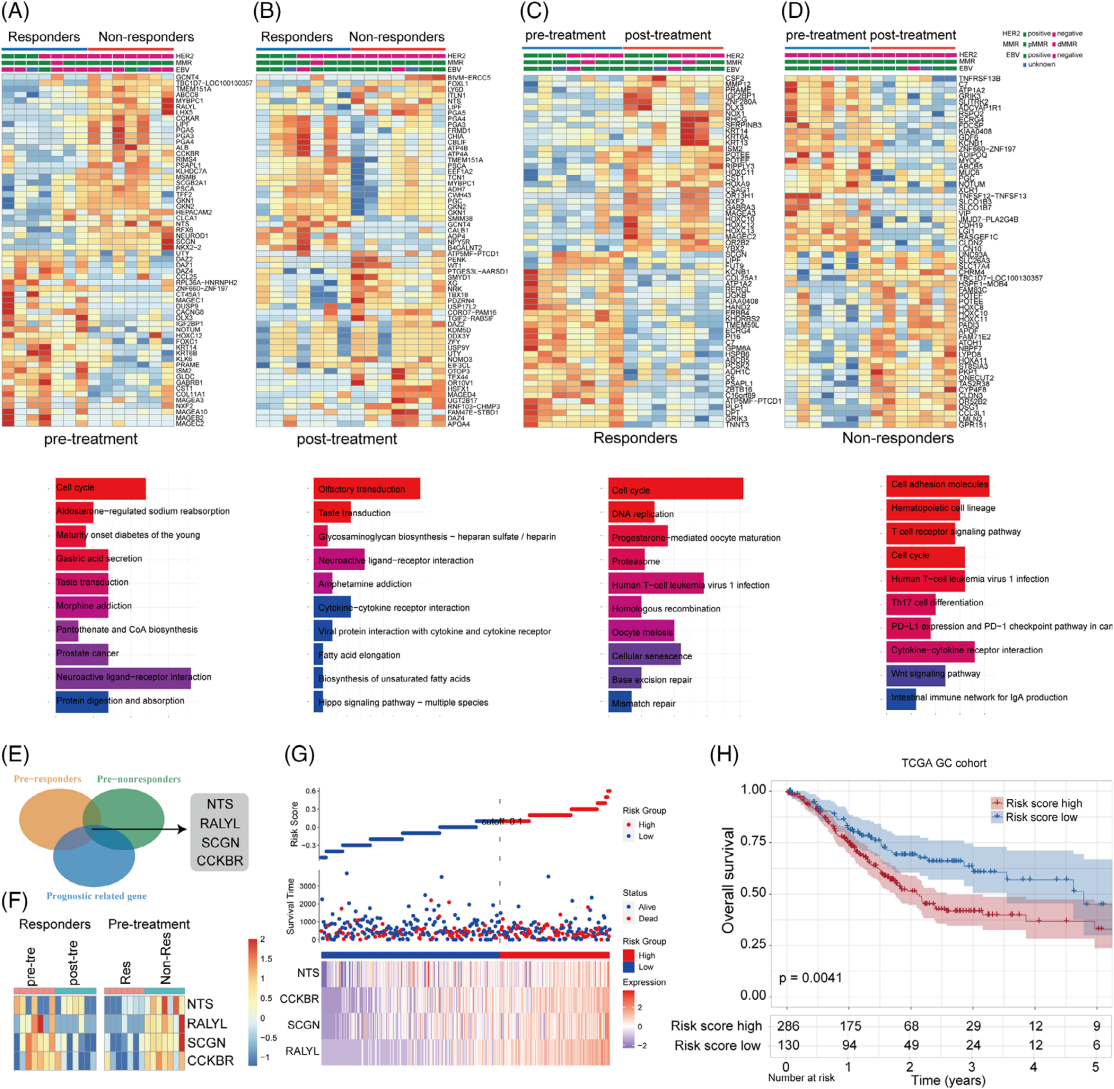
Transcriptome analysis and changes during neoadjuvant therapy. Heatmap showing DEGs between responders and non‐responders from pre‐ (A) and post‐treatment (B) biopsies, pre‐ and post‐treatment from responders (C) and non‐responders (D). KEGG function enrichment analysis is shown in panel below. (E) Venn diagrams showing the overlap in genes significantly changed in the responders group and non‐responders group pre‐ and post‐treatment. (F) Heatmap showing DEGs between pre‐ and post‐treatment and responders and non‐responders from pre‐treatment. (G) Risk curves and scatter plots for candidate risk genes in the TCGA‐GC cohort. (H) Kaplan–Meier analysis showing overall survival in low‐risk and high‐risk patient groups in the TCGA‐GC cohort.

In addition to investigating biomarkers that predict pathological response by comparing baseline responders and non‐responders, we also examined molecular events that trigger pathological response or induce treatment resistance. This was achieved by comparing responders before and after treatment and comparing non‐responders in the same manner. RNA‐seq was performed on baseline and resection samples from 14 patients (seven responders and seven non‐responders). Initially, we analyzed genes that exhibited differential expression (DEGs) in both groups prior to and following treatment (Figure 5A–D). The findings showed that the responder groups, which demonstrated the most clinically beneficial differential pathways, primarily exhibited enrichment pathways associated with the cell cycle, DNA replication, cellular senescence, and mismatch repair (Figure 5A–D). Conversely, the non‐responder groups, which displayed the most significant distinct pathways with adverse clinical impact, were primarily associated with molecules involved in cell adhesion, hematopoietic cell lineage, T‐cell receptor signalling, Th17 cell differentiation, PD‐L1 expression, and the PD‐1 checkpoint pathway (Figure 5A–D).

**FIGURE 6:**
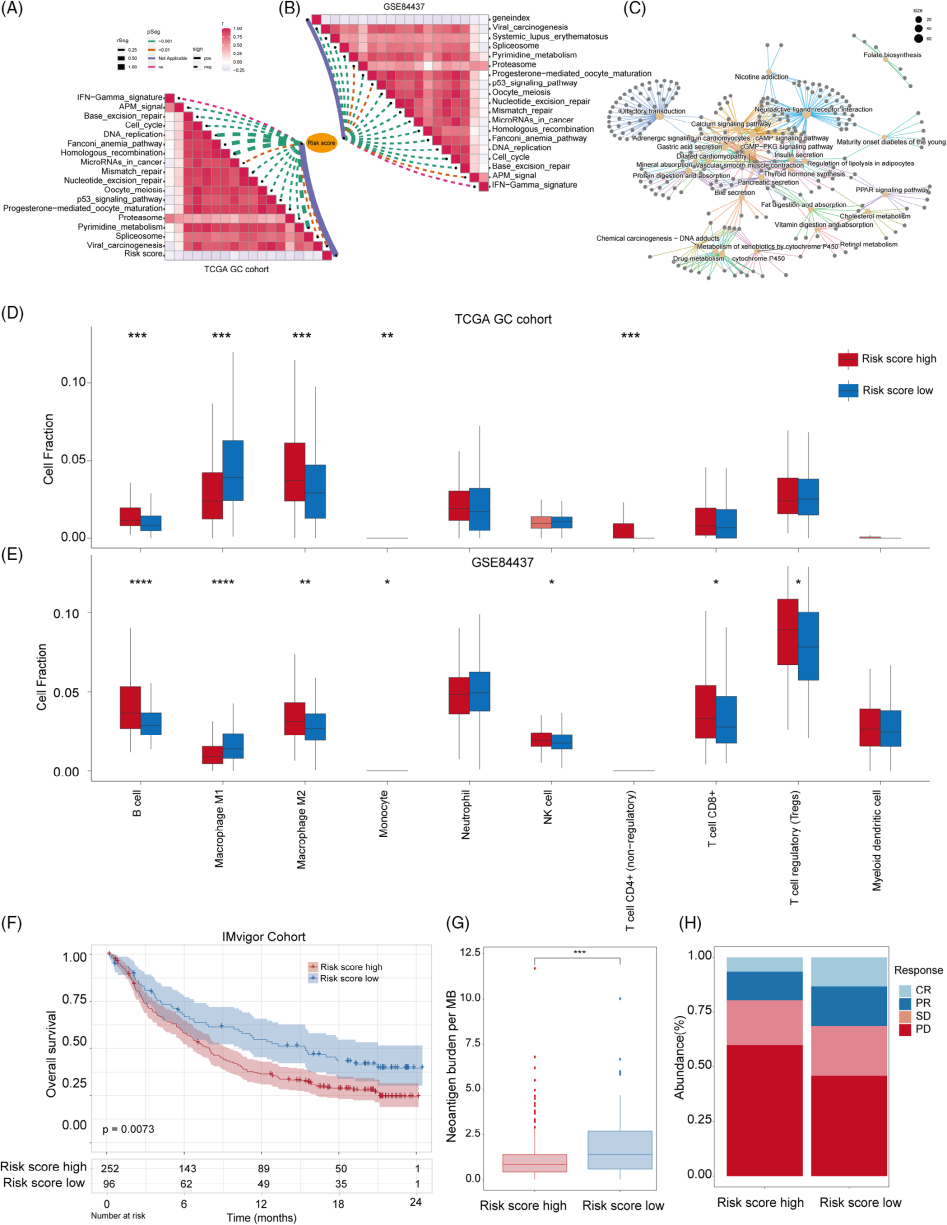
Roles of the risk model in predicting immune phenotypes. (A, B) Correlations between risk score and the enrichment scores of immunotherapy‐predicted pathways in two independent cohorts (TCGA‐GC and GSE84437). (C) GO analysis of the DEGs between low‐risk and high‐risk patient groups visualized by ClueGO. (D, E) Comparison of the estimated proportion of ten lymphocytes between low‐risk and high‐risk patient groups in two independent cohorts (TCGA‐GC and GSE84437), respectively. (F) Kaplan–Meier curves show overall survival in the high‐risk and low‐risk subgroups after the PD‐L1 blockade immunotherapy in the IMvigor210 cohort. (G) Differences in neoantigen burden between high‐risk and low‐risk subgroups in the IMvigor210 cohort. (H) The proportion of patients in the IMvigor210 cohort with different responses to PD‐L1 blockade immunotherapy.

Subsequently, four prognosis‐related differential genes (*RALYL*, *SCGN*, *CCKBR*, *NTS*) were identified based on both our expression profile cohort and the TCGA GC cohort (Figure 5E). In the responders, a substantial decrease in the expression levels of these four genes was noted prior to treatment. Conversely, the non‐responders exhibited significantly higher expression levels of these four genes compared with the responders group. (Figure 5F). Moreover, patients with low expression levels of *RALYL*, *SCGN*, *CCKBR*, and *NTS* exhibited longer survival in the TCGA GC cohort (Figure 5G). Using the median values obtained from ssGSEA scores of the four genes, individuals in the TCGA‐GC cohort were categorized into high‐ and low‐risk groups. The high‐risk group exhibited significantly lower OS rates, indicating the crucial clinical relevance and potential utility of this risk model as a cancer diagnostic biomarker (Figure 5H). The associations between the risk score and the expected immune checkpoint blocker response signatures were also examined. Our findings revealed a negative correlation between the risk score and the enrichment scores for all positive signatures related to immunotherapy (Figure 6A, B). According to the KEGG analysis, the genes exhibiting differential expression in the high‐ and low‐risk cohorts were primarily enriched in multiple pathways. These pathways encompassed neuroactive ligand‐receptor interaction, insulin signalling pathway, cGMP‐PKG signalling pathway, and cAMP signalling pathway (Figure 6C). Additionally, we analyzed the differences in immune cell infiltration between the two risk groups using ssGSEA and found Macrophage M2 was significantly higher, while Macrophage M1 levels were lower in high‐risk patients (Figure 6D–E). To summarize, the obtained results imply that immunotherapy response can be predicted by utilizing a risk signature comprised of four genes. Considering the observed correlation between the risk score and the immune microenvironment, we investigated the predictive capability of the risk score regarding immunotherapy response within the Imvigor210 cohort (Figure 6F). It was observed that patients with high‐risk scores in the anti‐PD‐1 cohort had worse prognoses. Additionally, the risk score was found to be lower in patients who responded positively to immunotherapy (PR and CR) and highest in patients who did not respond (PD) (Figure 6H). Additionally, the investigation assessed the burden of tumour neoantigens and revealed a reduction within the high‐risk score cohort when compared with those with a low‐risk score (Figure 6G). According to this discovery, it is implied that the decreased load of tumour neoantigens could be a potential factor in the group with high‐risk scores, leading to a disadvantage in survival and resistance to immunotherapy.

### 3.6 Stimulated immune infiltration responsible for drug resistance during neoadjuvant treatment

**FIGURE 7:**
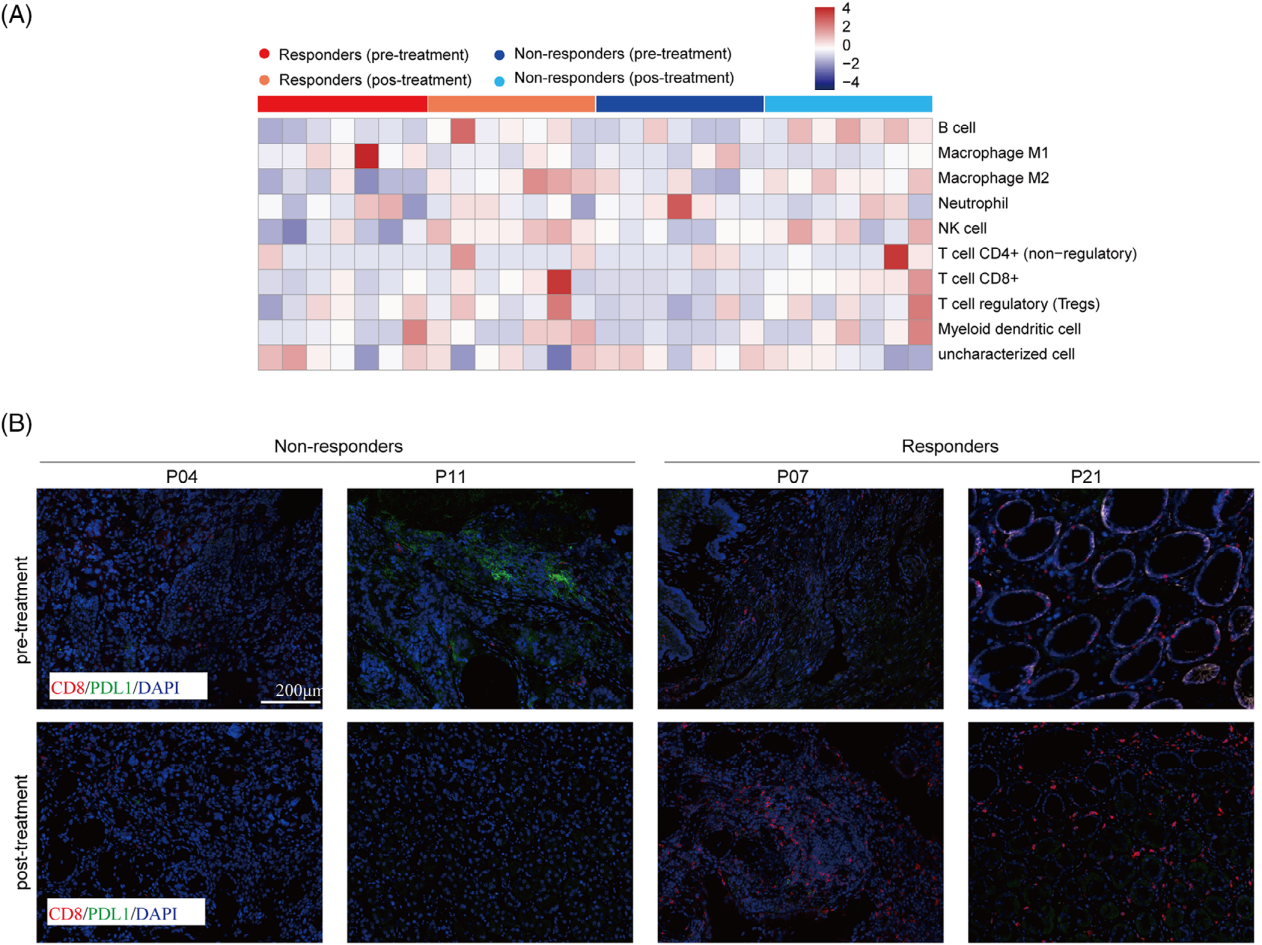
Immune cell subpopulation analysis. (A) Comparison of immune cell subpopulations assessed by the Quantiseq method using the RNA sequencing data including responders (*n* = 7) and non‐responders (*n* = 7). (B) Comparison of CD8 T cells in responders and non‐responders by immunofluorescence staining.

Next, we calculate the immune cell infiltration by comparing responders and non‐responders at pre‐ and post‐treatment to identify potential mechanisms of pathological response. Consistent with the above findings based on public datasets, higher Macrophage M2 and lower Macrophage M1 levels were observed in non‐responders at pre‐treatment (Figure 7A). In order to emphasize the influence of neoadjuvant therapy on the tumour microenvironment, mIF was utilized to evaluate the quantities of CD8 T cells and PD‐L1 cells. The findings exhibited a noteworthy rise in the number of CD8 T lymphocytes among individuals who exhibited favourable responses to the prescribed treatment. (Figure 7B). Taken together, these findings indicate that stimulated immune infiltration following chemoimmunotherapy is responsible for the therapeutic response.

## 4 DISCUSSION

Immunotherapy combined with chemotherapy has exhibited obvious efficacy benefits in treating advanced unresectable GC. Thus, there is an urgency to determine whether neoadjuvant treatment regimens enhance therapeutic responses in locally advanced GC. In this single‐arm phase II study of patients with locally advanced GC, neoadjuvant camrelizumab plus mFOLFOX6 exhibited a pCR rate of 9.1% and a near pCR rate of 20.0% in the surgical population set. Although the addition of camrelizumab did not demonstrate superiority in pCR rate improvement compared with other classical therapeutic regimens,^4^, ^5^, ^25^ both PFS and OS were increased (Table S2). The estimated 2‐year PFS and OS rates were 68% and 80%, respectively. Moreover, this trial demonstrated a favourable safety profile and feasibility. All patients completed the planned four cycles of neoadjuvant chemoimmunotherapy. The safety profile of camrelizumab combination therapy aligned well with that of mFOLFOX6 alone, without increasing toxicity or compromising surgical outcomes. No serious treatment‐related adverse events were observed in any of the patients. These findings underscore that the combination of camrelizumab and the less toxic mFOLFOX6 chemotherapy regimen as a neoadjuvant treatment is both safe and well‐tolerated.

Additionally, we conducted an analysis focusing on prognostic biomarkers and the dynamics of the tumour microenvironment during neoadjuvant treatment. The results indicated a correlation between changes in mRNA expression profile and treatment response. Neoadjuvant therapy was found to modify the tumour microenvironment and enhance the infiltration of CD8 T lymphocytes. Furthermore, patients with HER2‐positive and CTNNB1 mutations exhibited higher response rates and longer survival times. Moreover, we established a risk model based on mRNA expression that could further differentiate between responders and non‐responders.

Several single‐arm exploratory trials of neoadjuvant chemoimmunotherapy with small sample sizes have reported their initial results, showing promising pCR rates. For instance, in a study of 24 patients receiving camrelizumab plus FLOT or FLOT alone, more patients responded to the combination treatment compared with chemotherapy alone, although none achieved a pCR.^9^ Similarly, sintilimab plus XELOX demonstrated a pCR rate of 19.4%.^10^ It is worth noting that the histologic signet‐ring cell type was associated with a poor prognosis.^26^ 10% of the patients (6/60) in this study had a histologic signet‐ring cell type, while in the sintilimab plus XELOX study, approximately 3% (1/36) had this type. Furthermore, pCR was defined using Becker's criteria, which require no residual tumour in the tumour bed.^27^ This definition was used in the neoadjuvant sintilimab plus XELOX trial and FLOT4 trials.^10^, ^28^ The randomized controlled phase III KEYNOTE‐585 trial enrolled locally advanced GC patients to receive chemotherapy plus pembrolizumab or placebo as neoadjuvant treatment. This study achieved a pCR rate of 12.9% in the main cohort and 13.0% in the main cohort+FLOT cohort of pembrolizumab group. Nevertheless, no statistical difference in event‐free survival (EFS) between the two groups whether in the main cohort (mEFS: 44.4 in pembrolizumab group vs 25.3 in the placebo group) or the main cohort+FLOT cohort (mEFS:45.8 in pembrolizumab group vs 25.7 in the placebo group).^29^Overall, these findings suggest that neoadjuvant chemoimmunotherapy is effective for patients with locally advanced GC.

Since GC is known for its heterogeneity, there is a pressing need to identify patients who can benefit from neoadjuvant immunotherapy. In our study, we observed a positive response to neoadjuvant therapy in 71.4% of patients with HER2‐positive GC. Furthermore, the objective response rate was considerably enhanced in HER2‐positive unresectable advanced GC through the incorporation of pembrolizumab alongside trastuzumab and chemotherapy, as evidenced by the KEYNOTE‐811 clinical trial.^30^ These findings further support the use of neoadjuvant chemoimmunotherapy as the preferred treatment option for HER2‐positive resectable locally advanced GC. Moreover, genomic and transcriptomic analysis in our study revealed the involvement of the WNT/β‐catenin pathway at baseline. A pre‐clinical study in melanoma and a clinical study of 34 patients with hepatocellular carcinoma showed that the active WNT/β‐catenin pathway can lead to resistance to PD‐1 inhibitors.^31^, ^32^ However, in 73 metastatic melanoma samples, no association was found between the WNT/β‐catenin pathway and treatment response.^33^ In this study, CTNNB1 mutations p.D32N, p.S47C, and p.S45C were detected in three responders. The baseline WNT/β‐catenin pathway was enriched in responders compared with non‐responders and in post‐treatment non‐responders compared with pre‐treatment. This finding indicated that the WNT/β‐catenin pathway might contribute to immunotherapy response, however, which needs further exploration. Transcriptomic analysis revealed cell cycle‐related pathways and immune regulatory pathways significantly altered between responders and non‐responders. Moreover, we also constructed a prediction risk model based on *RALYL*, *SCGN*, *CCKBR*, and *NTS* mRNA expression. Previous investigations have shown that the prediction of effectiveness and prognosis cannot be reliably achieved using a single biomarker.^34^, ^35^ The current approach, therefore, involves combining multiple markers, However, it is important to validate this deduction through further verification. It is noteworthy that patients in the high‐risk group were associated with a worse prognosis in the TCGA cohort, while Macrophage M2 and Treg cells were significantly higher in patients with high risk. Accumulated evidence has established a role for Macrophage M2 and Treg cells in promoting immune escape. This suggests that our neoadjuvant immunotherapy regimen may function by negatively regulating macrophage M2 and Tregs cells. Our finding indicating macrophage M1/M2 polarization remarkedly affects immune checkpoint therapeutic response in GC, which needs further exploration. Recently clinical trials have shown promising results for neoadjuvant treatment in GC using various therapeutic approaches and ICIs. These studies, often exploratory with small sample sizes, have utilized multi‐omics to discover novel biomarkers.^36^, ^37^, ^38^ Gene mutations, specifically RREB1 and SSPO, along with TMB, have been linked to positive outcomes in patients treated with a PD‐1 inhibitor, apatinib, and chemotherapy.^37^ Comparable observations regarding TMB were made in a neoadjuvant trial involving camrelizumab combined with chemoradiotherapy for GC.^36^ Additionally, the presence of CD8+ T cells and a higher ratio of M1 to M2 macrophages were associated with better pathological responses,^37^, ^38^ findings that align with our research.

This study has some limitations, including its exploratory nature, small sample size, lack of a control group, and short post‐operative follow‐up period. The focus of this study is on biomarkers predicting pathological response, which differs from findings in current phase II trials of neoadjuvant immunotherapy for GC. However, our findings reveal various molecular factors associated with immunotherapy response. Notably, the incorporation of genetic instability and gene expression categories has identified distinct patient clusters with varying response rates. If these observations are validated in future trials, they could help identify patients likely to respond to treatment and reduce unnecessary treatment. Prospective validation is currently ongoing (NCT05583383).

## 5 CONCLUSION

In conclusion, neoadjuvant treatment with a PD‐1 inhibitor (camrelizumab) plus mFOLFOX6 demonstrated preliminary efficacy and extended survival in patients with locally advanced GC. The baseline HER2‐positive status and CTNNB1 mutations were correlated with treatment sensitivity and a favourable prognosis. Additionally, the infiltration of CD8+ T lymphocytes post‐treatment positively correlated with pathological response. Due to the small cohort, the preliminary findings of this study require further confirmation and validation.

## AUTHOR CONTRIBUTIONS

Ying Liu and Yuzhou Zhao conceived and designed the study. Jing Zhuang, Zhimeng Li, Zhi Li, Danyang Li, Jinbang Wang, Yong Zhang, Ke Li, Shuning Xu, Sen Li, Pengfei Ma, Yanghui Cao, Chenyu Liu, and Lei Qiao enrolled the patients and collected the data. Qingxin Xia, Juan Yu, and Chengjuan Zhang collected the biopsies. Chunmiao Xu was responsible for imaging evaluation.Ying Liu, Danyang Li, and Du Wang directed the statistical analysis. All authors participated in data interpretation. Zhentian Liu, Jinwang Wei, and Xuan Gao were responsible for bioinformatics analysis. The manuscript was prepared, reviewed, and revised by all authors. The final version to be submitted was approved by all authors.

## CONFLICT OF INTEREST STATEMENT

Zhentian Liu and Xuan Gao were employees of Geneplus. Jinwang Wei was an employee of GenomiCare Biotechnology LA Co., Ltd.(Shanghai). Zhiguo Hou, Chenxuan Liu, Rongrong Zheng and Du Wang were employees of Jiangsu Hengrui Pharmaceuticals. The remaining authors declare no conflict of interest.

## FUNDING INFORMATION

This study was funded by Jiangsu Hengrui Pharmaceuticals and the Innovation Team of Affiliated Cancer Hospital of Zhengzhou University.

## ETHICS APPROVAL STATEMENT

The study protocol and all amendments were reviewed and approved by the Institutional Review Board and Ethics Committee of Henan Cancer Hospital (ID:2019209). This study was conducted in accordance with the Good Clinical Practice Guidelines of the International Conference of Harmonization and the principles of the Declaration of Helsinki.

## PATIENT CONSENT STATEMENT

All patients provided written informed consent for participation and sample collection before enrollment.

## CLINICAL TRIAL REGISTRATION

Clinical trial information: NCT03939962.

## Supporting information

Supporting Information

## Data Availability

The genomic and transcriptomic data supporting this study's findings are available from the website (https://ngdc.cncb.ac.cn/gsa‐human/; Accession codes: HRA002739, HRA002845, HRA002829). The clinical trial data are available from the corresponding author and the Ethics Committee of Henan Cancer Hospital upon reasonable request. Further information and requests for resources and reagents should be directed to and will be fulfilled by the corresponding author.
