# Supplementary material for: Comprehensive multi‐omics analysis of resectable locally advanced gastric cancer: Assessing response to neoadjuvant camrelizumab and chemotherapy in a single‐center, open‐label, single‐arm phase II trial
Source: Clin Transl Med. 2024 Apr 29;14(5):e1674. doi: 10.1002/ctm2.1674 (PMC11058238; doi:10.1002/ctm2.1674)
Supplement: Supplementary file 1 — Supporting Information [file CTM2-14-e1674-s001.docx]

**Comprehensive Multi-Omics Analysis of Resectable Locally Advanced Gastric Cancer: Assessing Response to Neoadjuvant Camrelizumab and Chemotherapy in a Single-Center, Open-Label, Single-Arm Phase II Trial**

Yuzhou Zhao^1^*, Danyang Li^2^*, Jing Zhuang^1^*, Zhimeng Li^1^, Qingxin Xia^3^, Zhi Li^1^,Juan Yu^4^, Jinbang Wang^1^, Yong Zhang^5^, Ke Li^2^, Shuning Xu^2^, Sen Li^1^, Pengfei Ma^1^, Yanghui Cao^1^, Chenyu Liu^1^, Chunmiao Xu^6^, Zhentian Liu^7^, Jinwang Wei^8^, Chengjuan Zhang^4^, Lei Qiao^2^, Xuan Gao^7^, Zhiguo Hou^9^, Chenxuan Liu^9^, Rongrong Zheng^9^, Du Wang^9^, Ying Liu^2^†

**Supplement Methods**

**WES**

WES was performed on tumor samples from 23 patients (eight responders and 15 non-responders). Paired peripheral blood lymphocytes were used as germline control samples. DNA from formalin-fixed paraffin-embedded (FFPE) tissues and peripheral blood lymphocytes was extracted using a DNeasy Blood & Tissue Kit (Qiagen, Hilden, Germany). DNA concentration was measured using a Qubit fluorometer dsDNA HS kit (Invitrogen, Carlsbad, CA, USA). The library was constructed using a custom 53 M length capturing probe (Integrated DNA Technologies, IA, USA). Captured libraries were then pair-end sequenced in 100 bp lengths with a GenePlus-2000 sequencing platform (GenePlus, Beijing, China) following the manufacturer's guidance. Raw data were filtered to remove low-quality reads and adaptor sequences. Clean reads were mapped to the reference human genome (HG19) for mutation calling using a BWA aligner (version 0.7.10).

**Somatic mutation calling**

MuTect[1] was used to identify somatic single nucleotide variants (SNVs). Pindel [2] was used for somatic small insertions and deletions (INDELs). Mutations previously reported in the public database (dbSNP138, 1000Genomes, ESP6500, and EXAC) with > 1% allele frequency were removed. Mutation filtering criteria were: (i) sequencing depth ≥ 50 for tumor and ≥ 30 for normal, (Ⅱ) tumor allele frequency ≥ 5% for SNVs and ≥ 10% for INDELs, and (Ⅲ) normal allele frequency < 1%.

**Somatic copy number aberration analysis**

For comparison of SCNAs between samples, ExomeCN, a gene-based SCNA analysis algorithm, was applied. Specifically, we first obtained copy number segments with copy ratios between tumor and normal. The log_2_ copy number ratios of the segments were then assigned to the genes within the segments by CNTools, so each sample would have log_2_ ratio values of same number of genes for a fair comparison between samples. We defined copy number gains and losses in all tumor samples using +log_2_1.5 for gain and -log_2_1.5 for loss, respectively. Since the signal to noise ratio of SCNA could be reduced in the samples with lower tumor purity, we obtained purity-adjusted log_2_ ratios by log_2_((original copy ratio − 1)/purity + 1) if any of the paired samples from the same patients passed the original log_2_ thresholds of +log_2_1.5 and −log_2_1.5. Tumor purity was estimated by Sequenza. Copy number gain burden and loss burden were defined as the number of copy number gains and losses in a given sample.

**RNA extraction and sequencing**

RNA sequencing was performed on paired pre and post-treatment tissues from 16 patients (seven responders and nine non-responders). Total RNA was extracted from FFPE tissues using TRIzol (Invitrogen, USA) according to manufacturer’s instructions and quantified with the NanoDrop spectrophotometer (NanoDrop, USA). After rRNA removal with an Epicentre Ribo-Zero kit (Illumina, USA), mRNA sequencing library was constructed according to the protocols of TruSeq Stranded Total RNA Library Prep Kit (Illumina, USA) and evaluated with Agilent 2100 Bioanalyzer (Agilent Technologies, USA). RNA sequencing was performed with paired-end 2 × 150 on the Illumina Hiseq 2500 platform (Illumina, USA).

**Reference**

1. Cibulskis, K., et al., *Sensitive detection of somatic point mutations in impure and heterogeneous cancer samples.* Nat Biotechnol, 2013. **31**(3): p. 213-9.

2. Ye, K., et al., *Pindel: a pattern growth approach to detect break points of large deletions and medium sized insertions from paired-end short reads.* Bioinformatics, 2009. **25**(21): p. 2865-71.

**FigureS1:**

**Copy number variation analysis in patients grouped by pathological tumor response.**

Responder: pathological complete response (pCR) or near pCR; non-responder: partial response or poor or no response.

**Table S1** **Adjuvant treatment.**

|  | **Total**  **(n= 52)** | **Responder**  **(n = 16)** | **Non-responder**  **(n = 36)** |
| --- | --- | --- | --- |
| **Median cycles of adjuvant treatment** |  | 12 (4-14) | 10 (2-13) |
| **Treatment discontinuation, n (%)** |  |  |  |
| Unacceptable toxicity |  | 1 (6.25) | 1 (2.8) |
| Disease progression |  | 0 | 7 (19.4) |
| Patient’s choice |  | 1 (6.25) | 0 |

**Table S2** **A summary of the studies of neoadjuvant chemotherapy in locally advanced gastric cancer discussed in this study**

| **Trial** | **Design** | **Perioperative treatment** | **Patients** | **cT4** | **Two years survival** | | **Country** |
| --- | --- | --- | --- | --- | --- | --- | --- |
|  |  |  |  |  | **DFS/PFS rate** | **OS rate** |  |
| RESOLVE | Phase Ⅲ RCT | SOX | mITT: 337 | 97% (327/337) | 65% | 77% | China |
| FOCUS | Phase Ⅲ RCT | SOX vs FOLFOX | mITT: 288 vs 283 | NA | 71% | 78% | China |
| FLOT4 | Phase Ⅲ RCT | FLOT vs ECF/ECX | 356 vs 360 | 8% (28/356) vs 9% (33/360) | 54% | 68% | German |
| This study | Phase Ⅱ single-arm | FOLFOX | 60 | 46.7% (28/60) | 60% | 80% | China |
